# Commercial influences on patient and public involvement: a renewed call for research and action

**DOI:** 10.1093/heapro/daae188

**Published:** 2024-12-09

**Authors:** Marita Hennessy, Tom Fahey, James Larkin

**Affiliations:** College of Medicine and Health, University College Cork, Cork, Ireland; Department of General Practice, RCSI University of Medicine and Health Sciences, Dublin, Ireland; Department of General Practice, RCSI University of Medicine and Health Sciences, Dublin, Ireland

**Keywords:** conflict of interest, ethics, patient advocacy, patient and public involvement, pharmaceutical industry, public health, research

## Abstract

Patient and public involvement is increasingly advocated in health policy, research and practice. Patients and people with lived experience, carers and the general public should have a say in how policy is generated, how services are delivered and how research is conducted. Through this perspective article, we hope to stimulate discussion and debate around industry influence in patient and public involvement, specifically pertaining to patient organizations, which often play a key role in patient and public involvement activities. As momentum gathers around patient and public involvement in many countries, it is timely to discuss the nature and extent of commercial influences in such activities, the (un)anticipated consequences of industry–patient interactions, including conflicts of interest and motivated bias, and how we might better manage, or negate, such interactions. Patient and public involvement must be integral to research, policy and practice. While further research is needed to examine the interactions, and consequences of pharmaceutical industry interactions with patients, several practical steps can be taken in the interim. Structures, processes and supports, which are fit for purpose, are needed to ensure independence, power and legitimacy within patient and public involvement activities, and that patient advocates have their voices heard, and ultimately acted upon.

**Contribution to Health Promotion**

- Outlines evidence on the types of interactions between industry and patient organizations, the perceived benefits, and the concerns regarding undue industry influence
- Highlights the need to ensure independence, power and legitimacy of patient and public involvement activities.
- Calls for more research on industry–patient interactions, including the nature, extent and impacts of such interactions, as well as strategies to mitigate or manage conflicts of interest, as part of efforts to address the undue influence of commercial actors more broadly.

## INTRODUCTION

Patient and public involvement is increasingly advocated in health policy, research, and practice. It is vital, and indeed a democratic right, as people who use services, carers and the public should have a say in how services are delivered, as emphasized in the Declaration of Alma Ata (World Health Organization, 1978). Patient and public involvement in research specifically entails research being carried out ‘with’ or ‘by’ members of the public, rather than ‘to’, ‘about’ or ‘for’ them (NIHR, 2021). It can mean public or patient involvement at all stages of the research process, with the spectrum of involvement ranging from consultation at discrete points to members of the public and/or patients initiating and leading the research process from the outset. Patient and public involvement can enhance research quality and appropriateness, providing researchers with greater insights into the topic and outcomes of interest, and enhancing both recruitment rates and study design (Ennis and Wykes, 2013; Brett *et al.*, 2014; Domecq *et al.*, 2014). The purpose of this article is not to detail the merits of patient and public involvement—in research, policy or practice, we start our arguments from this position of value. We also wish to acknowledge at this point that while ‘patient and public involvement’ is a relatively recent concept in certain parts of the world (including Ireland where the authors are based), and particularly in relation to clinical and health services research, other participatory approaches—such as community-based participatory research—have been in use for decades within the field of health promotion and beyond (Gilfoyle *et al.*, 2022).

While much has been written on power dynamics within patient and public involvement, specifically between patients or members of the public and professionals and/or researchers (Locock *et al.*, 2017; O’Shea *et al*., 2019), less attention has been paid to other power relations within patient and public involvement, namely, those regarding industry or commercial influences. Patients and patient organizations play important roles in policy (Baggott and Jones, 2018) and research (Lexchin *et al.*, 2022) processes within many countries internationally, and independence from commercial influence is needed (Lexchin *et al.*, 2022). Patient and public involvement endeavours can be led by various actors, with and/or without direct or indirect commercial or industry influence(s), including—but not limited to—public bodies or publicly-funded researchers, public-private partnerships, or industry or industry-funded researchers. As momentum increases around patient and public involvement in research specifically, we believe that it is timely to discuss the extent and role of industry in involvement activities, the (un)intended consequences of industry–patient interactions and associated conflicts of interest, and how we might better manage, or mitigate, such interactions. Conflicts of interest are defined as *circumstances that create a risk that professional judgments or actions regarding a primary interest will be unduly influenced by a secondary interest* (Institute of Medicine (US) Committee on Conflict of Interest in Medical Research, Education, and Practice, 2009). It is important that patient organizations keep their primary interests—those of patients and carers, and at times the wider public interest—to the fore in their activities and actions (Baggott and Jones, 2018). Underpinned by social justice, patient organizations are guided by the principles of justice, beneficence and empowerment (Müller *et al.*, 2021). Interactions with industry, through receipt of industry funding or training, for example, can challenge their credibility, legitimacy and independence. Through this perspective article, we hope to stimulate discussion and debate in order to enhance policy and practice in this area. Firstly, we discuss the general issues arising from industry–patient interactions more broadly and then focus on patient and public involvement. We then discuss ways to minimize and manage industry influence on patient and public involvement activities.

Before we examine these issues, we wish to acknowledge the varied terminology used to describe people who engage in health(care)-related advocacy and/or research (e.g. ‘patients’, ‘consumers’, ‘services users’, ‘survivors’, ‘carers’) and how this occurs (e.g. ‘involvement’, ‘engagement’, participation, ‘co-design’, ‘co-production’). In this article, we use the term ‘patient and public involvement’, consistent with policy and practice developments in Ireland and the UK. We use the term ‘patient advocates/advocacy’ to reflect broader advocacy efforts, including but not limited to patient and public involvement activities. Where possible, we use the terms involvement and engagement when referring to patient and public involvement/engagement, and use the term interactions when referring to more broader relations or activities between industry and patient organizations. Various terms are used within the literature to describe organizations that aim to advocate on behalf of, represent and support, a group of people affected by a particular condition or conditions; they include patients’ organizations/associations/groups, patient advocacy organizations, patient–consumer organizations, and health consumer and patients’ organizations (Baggott and Jones, 2018). In this article, we use ‘patient organization’ throughout to describe such organizations or groups. We define patient organizations in line with Jones’s (2008, p. 930) definition of health consumer groups: ‘voluntary sector organisations that promote and/or represent the interests of patients, users and carers’; they can provide self-help, advice and advocacy. While this perspective piece has relevance to individual patients, our lens tends to focus on patient organizations. It is more common for public and private health services and research institutions to work with patient organizations rather than with directly recruited groups of patients or members of the public, as observed in a 2020 systematic review (which was supported by in-kind contributions of the European Federation of Pharmaceutical Industries and Association) (Biddle *et al.*, 2021). Finally, we appreciate that the term ‘patient’ is not unproblematic, e.g. not all people who use health services are, or identify as, ‘patients’ (Costa *et al.*, 2019); we use the term broadly.

## INDUSTRY–PATIENT INTERACTIONS: HAVE WE CAUSE FOR CONCERN?

Pharmaceutical companies are increasingly interacting with, and investing in, patients (Sheridan, 2018; Batt *et al*., 2020; Butler and Fugh-Berman, 2020) through, for example, training and networking opportunities and/or funding of patient organizations (Butler and Fugh-Berman, 2020), as well as current or former employees holding positions on governing boards of such organizations (McCoy *et al.*, 2017). Through the creation of tools and frameworks to involve patients in research and development, the pharmaceutical industry is also actively moulding the landscape of patient involvement in drug development (Zvonareva, 2023). Pharmaceutical industry funding of patient organizations is common (O’Donovan, 2007; Hemminki *et al*., 2010; Fabbri *et al*., 2019, 2020; Mandeville *et al.*, 2019; Ozieranski *et al.*, 2019; Parker *et al.*, 2019; Batt *et al.*, 2020; Khabsa *et al.*, 2020; Lexchin *et al.*, 2022; Somers *et al.*, 2024); even normalized (Batt *et al.*, 2020). For example, in 2022, the International Alliance of Patients’ Organizations, whose membership includes hundreds of patient organizations, was almost entirely industry-funded (98%), namely by pharma (International Alliance of Patients’ Organizations, 2023a, 2023b). In the same year, two-thirds of the European Patients Forum’s funding came from industry (European Patients Forum, 2022). This culture of industry funding is perhaps unsurprising, given that there is a culture of industry sponsorship of health professionals and medical societies (Fabbri *et al.*, 2016; Boytchev, 2023). Indeed health professionals often have a role in establishing and supporting patient organizations (Baggott and Jones, 2014).

Patient organizations that accept industry funding participate in ‘asset exchange’ with companies, as observed in an interview study with people working in industry-funded patient organizations, including CEOs, other staff and board members (Parker *et al*., 2019). For example, pharmaceutical companies can provide (often much-needed) funding to patient organizations for various activities including educational events, research, disease awareness-raising campaigns, informational resources and training (Jones, 2008; Hemminki *et al.*, 2010; Colombo *et al.*, 2012; Parker *et al.*, 2019). Patient organizations on the other hand can provide the industry with a range of benefits including assistance with advocacy, relationship-building with key opinion leaders, support in raising disease awareness, assistance with trial recruitment and importantly they can provide the industry with credibility (Hemminki *et al.*, 2010; Ozieranski *et al.*, 2019; Parker *et al.*, 2019). They can also promote companies in various ways, for example, by having advertisements and/or web links on these companies’ websites (Colombo *et al.*, 2012). More recently, there has been a rise in ‘patient influencers’ on social media, who can have varied types of interactions with pharmaceutical companies, through paid and unpaid roles (Willis *et al.*, 2023). The pharmaceutical industry has indeed established itself as a legitimate partner of health advocates across the world (O’Donovan, 2007), and legitimacy is a key mechanism by which corporations exercise power (Lacy-Nichols and Marten, 2021).

Patient organizations are also powerful actors in policy, research and practice, however, though this may be constrained by the contexts within which they operate; they ‘have assets that the industry covets’, including the trust of patients and support of broader public (Batt, 2017, p. 283). The influence of pharmaceutical industry funding on health professionals and researchers is well-documented; there is evidence to suggest that patient advocates—as individual patients, and/or as part of patient organizations—are also susceptible to the bias caused by such interactions. Much attention has been drawn to the negative influence of pharmaceutical companies on the attitudes and practices of health professionals, through a variety of mechanisms, including funding, training, and other marketing strategies (DeJong *et al.*, 2016; Mintzes *et al.*, 2018; Menkes *et al.*, 2024). A systematic review found that receipt of industry payments is associated with increases in the prescribing of the paying company’s drug, prescribing costs, and prescribing of branded drugs over generics (Mitchell *et al.*, 2021). Research also demonstrates that the results of drug industry-funded studies tend to report in favour of their products (Lundh *et al.*, 2017). The overall consequences of this are lower quality care and higher costs to healthcare systems. Furthermore, these interactions, and the conflicts of interest they create, have undermined trust in research and the medical profession. There is evidence that similar interactions between patient organizations and industry are causing similar biases (Fabbri *et al.*, 2020); we discuss this more in the next paragraph. To rectify this situation and ensure the independence, power and legitimacy of patient and public involvement activities, the role of industry funding and interactions must be given detailed consideration. While there have long been concerns about industry influence on patients (Herxheimer, 2003; Boseley, 2006; Consumers International, 2006; O’Donovan, 2007; Baggott and Forster, 2008; Jones, 2008; Moynihan and Bero, 2017), we argue that these need to be brought into focus again and addressed, given the increasing emphasis on patient and public involvement in health research, policy and service delivery (Health Research Board, 2021; Vocal, MRC, 2023).

Despite the argument that pharmaceutical industry funding can enable patient organizations to provide useful services, the trade-offs must be considered. There is an inherent conflict between patients’ interests and those of pharmaceutical companies that have a fiduciary responsibility to their shareholders (Boatright, 1994). While it is critical to amplify patients’ voices, if the industry is funding patient organizations, then it calls into question: whose voice is being amplified? Some critics have gone as far as describing some patient organizations, along with some professional societies, as ‘front groups’ for industry (Marks, 2020). Patients may be more likely to adapt their views in line with the industry and be less likely to be critical of industry activities (including drug prices) when in receipt of industry funding (Batt *et al.*, 2020; Butler and Fugh-Berman, 2020). Patient organizations may find it difficult to maintain productive industry relationships while criticizing their industry partners at the same time (Jones, 2008). For example, a systematic review found that four studies which analysed the relationship between industry funding and the positions of patient organizations on a range of highly controversial issues found that industry-funded groups generally supported sponsors’ interests (Fabbri *et al.*, 2020). This is perhaps unsurprising given that the industry is more likely to fund patient groups that align with its priorities and product lines (Gentilini and Parvanova, 2023); we discuss this in more detail in the next section on industry influence. Acts, such as sponsorship, which build or deepen relationships between parties, can positively influence how the funded party perceives the funder and result in biased behaviours in the funder’s interests (Goldberg, 2019). Beyond this, industry funding of this nature creates epistemic corruption (Sismondo, 2021), whereby the funding creates greater space for industry-driven ideas, concepts and approaches. By funding patient organizations, the industry can legitimize these perspectives.

Examples of activities that patient organizations carry out—that it could be argued are primarily in the interests of industry and not necessarily in the interest of the patients they represent—include efforts to achieve greater acceptance of conditions as medical diseases (O’Donovan, 2007; Moynihan and Bero, 2017). Patients can also lobby health services and/or governments for access to certain treatments or medications which may not yet be approved for use, or to accelerate the approval process, with questions raised about the influence of pharmaceutical companies on such activities (Burton, 2005; Lewis, 2006; ThirdSector, 2006; Williams *et al.*, 2011; Bordogna, 2014; Roland, 2020; Largent *et al.*, 2021; Batt, 2023). In their study, Holman and Geisler (2018) observed how, following a series of failed approval attempts, a drug for female sexual dysfunction ultimately gained FDA approval arising from industry-affiliated patient involvement. Testimonies during the approval process differed between participants (patients) with industry affiliations and those without, with the former characterizing the issue in biological terms and their own success with pharmaceutical treatments (ibid). Industry funding of patient organizations that contribute to health technology (including drug) assessment processes (of their products, or of a competitor’s) within countries such as the UK (Mandeville *et al.*, 2019; Gentilini and Parvanova, 2023; Parvanova *et al*., 2023) and Canada (Lexchin, 2019) is highly prevalent; there are several examples of patient organizations not declaring their conflicts of interest when participating in these processes (Mandeville *et al.*, 2019). These industry-supported patient organizations’ activities, and indeed their awareness-raising of conditions rather than drugs, can provide pharmaceutical companies with an alternative way of ‘informing’ patients about their products as they are unable to market/advertise prescription drugs directly to patients—platforming industry interests (Herxheimer, 2003). Therefore, it is important to discuss industry funding of patient organizations and the bias that this may create in their patient and public involvement activities. These are important issues for patient organizations to consider, to ensure that they are accountable to—and are prioritizing the needs and views of—the (patients) they represent. Accusations of improper behaviour can cause reputational damage to patient organizations (Jones, 2008).

## INDUSTRY INFLUENCE ON PATIENT AND PUBLIC INVOLVEMENT ACTIVITIES

Examples of the types of patient and public involvement activities that the industry influences include research priority setting, informing the development of public policy or research policy, or directly informing research practice. This is not an exhaustive list. The motives for the industry to fund patient and public involvement activities are unlikely to be selfless. There is evidence to suggest that pharmaceutical companies interact more with, or will fund, existing patient groups if they have new drugs coming up for review before the government regulator for marketing approval and cost subsidy, or even provide seed funding to new patient groups when they have new products to market (Herxheimer, 2003; Parker *et al.*, 2019; Gentilini and Parvanova, 2023). Patient and public involvement activities are highly lucrative for pharmaceutical companies. For example, it has been reported that investing in patient and public involvement could accelerate a pre-phase 2 product launch by 2.5 years, or a pre-phase 3 by 1.5 years (Levitan *et al.*, 2018). Of further concern is that companies will prioritize investment in patient organizations—and as a consequence patient and public involvement activities—associated with certain conditions (for example, cancer and diabetes) based on their commercial viability (Ozieranski *et al.*, 2019; Mulinari *et al.*, 2020). It may also be the case that the industry will interact with certain types of patients or patient groups, and not with others, potentially amplifying a voice more aligned with its interests (Batt, 2017) and indirectly disempowering—or indeed silencing—other patient voices (Mulinari *et al.*, 2020). There are also examples of industry employing ‘corporate ventriloquism’ whereby it defers to the voices of industry-affiliated patients, masking its own influence over such discourses or individuals and/or groups (Holman and Geisler, 2018). Industry may also target patient groups where there is higher profitability, for example, rare diseases (Gentilini and Parvanova, 2023). Furthermore, industry co-funding of patient organizations and/or patient and public involvement initiatives that set research agendas through the hosting and funding of meetings/research prioritization exercises is problematic. Co-funders often include public bodies or funding agencies. For example, the Irish Platform for Patients Organisations, Science & Industry (IPPOSI), a leading actor in patient and public involvement activities—including research agenda-setting—in Ireland, receives two-thirds of its funding from industry, with the remainder from public sources, including the Irish Department of Health via the Health Research Board (ÌPPOSI, 2024). With the growth of patient and public involvement in research, these activities and practices have the potential to bias—and legitimize in the case of co-funding by public bodies—the research agenda, methodologies and research outcomes. This funding therefore has the potential to undermine trust in patient and public involvement which is an invaluable component of research, policy and practice.

Industry is also cognisant of criticisms surrounding its interactions with patients, citing several barriers to increasing such interactions, including negative media reporting and pharmaceutical industry codes of practice (Parsons *et al*., 2016). Some argue that the influence of pharmaceutical companies over the actions of patient organizations, while it has increased, has yet to be fully elucidated (O’Donovan, 2007). More research is needed to better understand the prevalence of industry funding and the effects of sponsorship (Colombo and Mosconi, 2020). We also suggest that interactions between industry and patient organizations are further explored to examine all potential mechanisms of influence, including those which may be slightly less visible, e.g. where industry actors have a governance role in patient organizations (McCoy *et al.*, 2017).

Patient groups themselves report different types of relationships with the pharmaceutical industry, and their views on these relationships also vary (Parker *et al.*, 2019). In an Australian study, the most common was a ‘successful business partnership’, with participants having close working relationships with industry staff; they acknowledged the potential for industry influence but reported confidence in strategies for avoiding this industry influence. Other relationships included ‘unsatisfactory’ (i.e. in receipt of industry money, but with misgivings/unease) or ‘undeveloped’ (i.e. not currently in receipt of funding, but potentially open to it), while some patients/patient groups felt their organizations’ missions were incompatible with those of the pharmaceutical industry (Parker *et al.*, 2019). In their study of online communication surrounding prescription medications, Willis *et al.* (Willis *et al.* 2023) found that patient influencers wanted to be an accurate, trustworthy source of information for their followers and did not want to work with a pharmaceutical company if it would restrict their messaging.

## MINIMIZING AND MANAGING INDUSTRY INFLUENCE ON PATIENT AND PUBLIC INVOLVEMENT ACTIVITIES

Some argue that concerns about industry interactions with patients are over-stated and that if patient organizations have robust governance around industry interactions then industry collaboration can be successful (Taylor and Denegri, 2017). Industry supporters also argue that we should not increase regulation of the pharmaceutical industry as it would risk ‘strangling the goose that lays the golden life-saving eggs that global health requires’ [(Freudenberg, 2014), p. 61]; an argument that is also frequently heard in relation to patient and public involvement—that advancements in relation to particular issues of importance to patients would not happen without industry support (Jones, 2008; Bruno and Rose, 2019). There are, however, several issues with such arguments.

Firstly, there can be a lack of ethical codes for cooperation with industry amongst patient groups (Hemminki *et al.*, 2010; Fabbri *et al.*, 2020). There is also a lack of transparency surrounding interactions between patient organizations and pharmaceutical companies (Hemminki *et al.*, 2010). Studies have found relatively high levels of non-disclosure of industry funding by patient organizations. For example, an Australian study found that 48% of a random sample of pharmaceutical industry-funded groups did not disclose their industry funding (Lau *et al.*, 2019). In their systematic review, Fabbri *et al.* (Fabbri *et al*. 2020) observed that 73% of patient organizations that received industry funding did not disclose this information on their websites. Furthermore, while there is some (albeit limited) disclosure of pharmaceutical industry funding by patient organizations, details are lacking including the specific financial values and activities supported, for example, on their websites and/or other publications (Herxheimer, 2003; O’Donovan, 2007; Jones, 2008; Colombo *et al.*, 2012; McCoy *et al.*, 2017; Batt *et al.*, 2020; Fabbri *et al.*, 2020; Lexchin *et al.*, 2022; Somers *et al.*, 2024). Similarly, disclosure of policies for governing industry interactions and managing conflicts of interest by patient organizations is also lacking (Herxheimer, 2003; Colombo *et al.*, 2012; McCoy *et al.*, 2017).

Industry often has its own codes in relation to interacting with patients; such codes exist in Ireland (Irish Pharmaceutical Healthcare Association, 2021) and the UK (Association of the British Pharmaceutical Industry, 2024), amongst other countries. The European Federation of Pharmaceutical Industries and Associations (EFPIA)—of which the Irish and UK bodies are members—Code of Practice on relationships between the pharmaceutical industry and patient organizations requires member companies to publicly disclose financial support and/or significant indirect or non-financial support to patients/patient organizations, including the nature of these relationships (EFPIA, 2020). The Code recommends providing data annually, on a national or European level, via reports/websites. While such information is made available, it is difficult to collate and interpret as reports are published on individual company websites (Ozieranski *et al.*, 2019). Furthermore, an analysis of payments disclosed on drug company and charity regulator websites noted that both pharmaceutical companies and patient organizations under-report financial payments of pharmaceutical companies to patient organizations (Ozieranski *et al.*, 2020). Payments to health professionals are more regulated. For example, in the US, under the Open Payments program established by the Sunshine Act (American Medical Association, 2020), manufacturers of drugs, medical devices and biologics are required to disclose a wide breadth of payments to physicians and teaching hospitals (Centers for Medicare & Medicaid Services, 2024). Some have suggested that patient advocacy groups should be brought under legislation such as the Sunshine Act and be mandated to disclose industry sponsorship (McCoy *et al.*, 2017; Moynihan and Bero, 2017; Fabbri *et al.*, 2020). An Australian study found that citizen health advocates had mixed feelings about accepting sponsorship from industry, but strongly supported mandatory transparency measures (Moynihan *et al.*, 2020). Over three-quarters of the participants in the aforementioned study by Willis *et al.* (Willis *et al.* 2023) agreed that transparency (regarding any industry interactions that they may have) was key. The lack of transparency in industry and patient organization reporting can heighten concerns regarding conflicts of interest and the perceived independence and legitimacy of patient groups (Jones, 2008; Baggott and Jones, 2018).

While greater transparency is needed, disclosure of funding alone is not enough (Rose, 2013; Bruno and Rose, 2019; Goldberg, 2019; Lexchin and Fugh-Berman, 2021). Indeed it may even have an unintended opposite effect, by creating a moral license and intensifying biased behaviours whereby the funding recipient believes that disclosure sufficiently mitigates any conflict of interest and therefore no further action is required to manage that conflict of interest (Goldberg, 2019). Individual patients and/or patient organizations—as with professionals/researchers—need to be conscious of the potential for ‘capture’ by commercial entities, and do everything possible to avoid them (Goldberg, 2019). The simplest solution is to not accept payments from the industry. This would however require alternative sources of funding from public monies and/or other non-conflicted sources. A hypothecated pharmaceutical industry tax could be considered, similar to that suggested for continuing medical education for healthcare professionals (Moriarty *et al.*, 2021). Such taxes however are not without criticism, with arguments that they can detract from more effective measures being implemented such as regulation and controls which could prevent harm from occurring in the first place (Schalkwyk *et al.*, 2023).

Many argue that such suggestions are too idealistic and that more realistic approaches are needed. Some argue, that to reduce the influence of industry, patient organizations should consider robust governance including: (i) having low limits on the amount of funding received from any one individual donor, (ii) transparency around the purpose and amount of industry funding and (iii) developing policies to manage conflicts of interests associated with industry funding (Taylor and Denegri, 2017). These policies could entail the receipt of unrestricted grants only, whereby the patient organization has complete autonomy over how to use the funding, and the sponsor has no say; however, unrestricted grants can also create unwanted biases (Fugh-Berman, 2021). There is also a potential role for charity regulators in implementing and enforcing processes and procedures to assist in the mitigation and/or management of conflicts of interest, where patient groups are registered charities; this warrants further exploration. With regard to patient and public involvement specifically, funders must create clear policies on industry activities in this area. For example, governmental and independent non-governmental research funding bodies should make reimbursement of patient and public involvement contributors—to a standard minimum rate—both a requirement and a component of their funding terms and conditions, thus limiting the need for industry support. Furthermore, conflicts of interest arising from industry-led or supported patient and public involvement-related education and training must also be acknowledged and mitigated; for example, in Europe, the European Patients’ Academy on Therapeutic Innovation (EUPATI)—a multi-stakeholder public-private partnership—provides education and training to patient advocates across member countries (EUPATI, 2024). Further work is needed to explore how to effectively manage conflicts of interest in industry–patient interactions, in patient and public involvement (in all its forms) and beyond.

As it is likely that—and similarly within the healthcare profession—some associated with patient organizations underestimate the potential negative effects of conflicts of interest, capacity-building amongst patient organizations is needed to foster further reflection, discussion and management of ethical and moral issues concerning industry interactions/funding, in addition to regulation and policy initiatives. Greater understanding of the power of corporations may help to identify actions to address it and thereby protect and promote public health (Wood *et al.*, 2022); similar issues apply to the power of corporations in industry–patient interactions, while interests may be perceived to align, industry holds much power which can negatively impact on patients/patient groups. While research around industry–patient interactions is growing, more is needed (Moynihan, 2020).

## THE PATIENT AND PUBLIC INVOLVEMENT IMPERATIVE

We cannot lose sight of the importance of patient and public involvement in research, policy and practice, and the need for such contributions to be adequately reimbursed or remunerated. Given deficits in state support for patients and patient organizations, it is unsurprising that they turn elsewhere (O’Donovan, 2007). Patient organizations can lack the financial and political resources to support their activities and missions (Jones, 2008; Baggott and Jones, 2014; Sienkiewicz and van Lingen, 2017). It is important to note that industry funding is often filling a gap that may go unfilled otherwise. This is particularly true for patients or members of the public who, unlike researchers in paid positions, may not have the security of a full-/part-time research position, but instead face many financial barriers to participation in patient and public involvement efforts. This is also relevant as there can be under-representation of certain groups or demographics within patient organizations also (Baggott and Jones, 2014, 2018). Beyond financial resources and supports, other costs to patient advocates—such as mental and emotional labour—should also be acknowledged and supported (Fuld Nasso *et al.*, 2021).

While pharmaceutical industry funding can assist patient organizations in achieving their missions, as highlighted above, it can have negative consequences. Patient(s)/patient organizations—and others—may see the immediate advantages of industry funding, without realising the broader implications associated with such relationships (Adams and Gregan, 2024). Patient organizations must consider the full implications of industry interactions as they are accountable for their actions, particularly to the patients they aim to represent (Müller *et al.*, 2021). Furthermore, as funders place more emphasis on patient and public involvement in research, they must also create clear policies on industry interactions in this area. Both funders and policymakers also need to develop mechanisms to provide adequate public funding for patient organizations.

## CONCLUSION

We call on policymakers, funders, researchers, practitioners, the public, and all those with an interest in involving members of the public and patients in research to engage with the issues relating to industry–patient interactions. Patient and public involvement in research, policy and the delivery of health care is paramount, as is the need to reimburse people’s time and contributions. While many support increased interactions between the pharmaceutical industry and patients in this regard, it is important to—at a minimum—acknowledge conflicts of interest and to put adequate, and independent, procedures in place to manage such conflicts. While further research is needed to examine the interactions and consequences of pharmaceutical industry interactions with patients through advocacy and patient and public involvement, several practical steps can be taken in the interim.

Independent, evidence-based training for patients and patient groups around interactions with industry and governance relating to assessing and managing conflicts of interest would be one such measure; this should be funded through public monies and/or suitable, non-conflicted, sources. Mandatory disclosure of industry funding of patient groups by both individuals, patient organizations, and industry (in an openly accessible format, ideally within one repository) would also be beneficial. Disclosure alone is insufficient however; every effort should be made to limit direct patient interactions with industry due to clear conflicts of interest. Where interactions cannot be avoided, for whatever reason, there should be limits to the direct contact that patients have with industry representatives. Governmental and independent non-governmental research funding bodies should make reimbursement of patient and public involvement contributors a requirement, thus limiting the need for industry interactions in the form of financing such arrangements. Patient and public involvement must be integral to health policy, research and practice. It is imperative that we have structures, processes and supports in place which are fit for purpose to ensure that patient and public involvement contributors have *their* voices heard, and ultimately acted upon.

## Data Availability

No new data were generated or analysed in support of this research.
